# Incorporation of tissue-based genomic biomarkers into localized prostate cancer clinics

**DOI:** 10.1186/s12916-016-0613-7

**Published:** 2016-04-04

**Authors:** Marco Moschini, Martin Spahn, Agostino Mattei, John Cheville, R. Jeffrey Karnes

**Affiliations:** Department of Urology, Mayo Clinic, Rochester, MN USA; Department of Urology, University Hospital of Bern, Inselspital, Bern, Switzerland; Klinik für Urologie, Luzerner Kantonsspital, Luzern, Switzerland; Department of Laboratory Medicine and Pathology, Mayo Clinic , Rochester, MN USA

**Keywords:** Prostate cancer, Radical prostatectomy, Genetic tools, Decipher, Oncotype DX, Prolaris

## Abstract

Localized prostate cancer (PCa) is a clinically heterogeneous disease, which presents with variability in patient outcomes within the same risk stratification (low, intermediate or high) and even within the same Gleason scores. Genomic tools have been developed with the purpose of stratifying patients affected by this disease to help physicians personalize therapies and follow-up schemes. This review focuses on these tissue-based tools. At present, four genomic tools are commercially available: Decipher™, Oncotype DX®, Prolaris® and ProMark®. Decipher™ is a tool based on 22 genes and evaluates the risk of adverse outcomes (metastasis) after radical prostatectomy (RP). Oncotype DX® is based on 17 genes and focuses on the ability to predict outcomes (adverse pathology) in very low-low and low-intermediate PCa patients, while Prolaris® is built on a panel of 46 genes and is validated to evaluate outcomes for patients at low risk as well as patients who are affected by high risk PCa and post-RP. Finally, ProMark® is based on a multiplexed proteomics assay and predicts PCa aggressiveness in patients found with similar features to Oncotype DX®. These biomarkers can be helpful for post-biopsy decision-making in low risk patients and post-radical prostatectomy in selected risk groups. Further studies are needed to investigate the clinical benefit of these new technologies, the financial ramifications and how they should be utilized in clinics.

## Background

Prostate cancer (PCa) represents the most commonly diagnosed internal malignancy among men in the Western world. In 2015, the number of men diagnosed with PCa was estimated to be over 220,000 in the United States alone [1]. The introduction of prostate-specific antigen (PSA) in the late 80s and the associated ‘stage migration’ have led to an increase of PCa diagnoses and specifically lower risk disease [2]. Although a number of these newly diagnosed patients are potentially eligible for active surveillance (AS), a large percentage still undergo active treatment given some uncertainty as to whether they harbor a ‘higher risk’ disease. Additionally, the number of patients diagnosed with high risk disease with an elevated risk of metastasis and death is still considerable, and an increasing trend has been described [3, 4]. In this setting, it is pivotal to increase our ability over-and-above routine clinicopathologic variables (T stage, PSA, Gleason scores and other cancer characteristics from biopsies and/or pathology specimens) to identify patients that could benefit from an active treatment after prostate biopsy, as well as patients that are at added risk for recurrence(s) after radical prostatectomy (RP). This is fundamental considering the potential morbidity associated with any active treatment, such as radiation and/or RP for PCa. None of the above represents novel concepts; however, there now exists biomarkers that may allow for better risk stratification to help decide on treatment versus no treatment. Patients can often be categorized into subgroups using the routine clinicopathologic parameters. However, better understanding of PCa biology using tissue-based biomarkers might help clinicians provide more ‘personalized’ treatments.

In this context, not only is the clinical behavior of PCa heterogeneous but so are the genomic alteration patterns within the biologic pathways, and several different mutations are needed during tumorigenesis to develop an aggressive PCa. For this reason, studying and cataloging these albeit quite complex alterations [5–7] has contributed to the need for development of complex biomarkers or genomic panels that are combined to improve prognostication of localized PCa, and more so to tailor both primary or secondary treatment and follow-up. Clinicians, most frequently urologists, are often challenged how to best advise newly diagnosed male patients with low-to-intermediate (3 + 4) risk prostate cancer, to undergo active surveillance versus active treatment, and patients are often left wanting more information to make that decision. Even after a PCa has been treated by a RP, patients with adverse pathology (non-organ-confined disease) or those that develop biochemical recurrence (BCR) have a disparate risk of developing metastasis, and secondary treatment, often radiation, is advocated. As alluded earlier, there are inherent limitations in the ability to prognosticate a potentially lethal cancer using pathology reports and PSA values. Genomic sequencing of advanced PCa highlights the complexity of this disease and its different states [8, 9]. In this context, biomarker panels or genomic tools have been developed for more localized disease that perhaps help to ‘simplify’ a response to a complex question, i.e. is there a lethal component of this hormone-sensitive PCa in the treatment-naïve state or post-RP? The information provided by these tools are presented as a risk percentage; for example, the percentage risk of upgrading from biopsy to a higher Gleason score, such as 4 + 3 at RP, or percentage chance of BCR or metastasis after RP. These tools do not provide a binary answer to treat or not to treat for the clinician or patient. They use archived formalin-fixed, paraffin-embedded (FFPE) tissue of either the highest volume tumor foci or Gleason score from either a biopsy or RP specimen. Each assay is unique. To date, there are no head-to-head studies evaluating one of these panels over the other when a comparison is relevant. Prior to these biomarker or genomic panels, there were no routine tissue-based biomarkers that were used in practice. As mentioned, the goal is early detection of a potentially ‘lethal’ PCa to treat and separation of this from a potentially indolent course. The development of metastasis is usually lethal despite survival gains by different systemic therapies [10–14]. Considering the vast research and data in this field, several previous reviews assessed the importance of tissue-based biomarkers [15–18]. In this context, we present an up-to-date overview of available tissue markers correlated with clinical utility in the field of PCa.

### Commercially available tissue-based prognostic biomarker panels

#### Decipher™

Decipher™ is a genomic test, co-developed by GenomeDx Biosciences (Vancouver, BC, Canada) and Mayo Clinic, to assess the probability of developing metastases after RP (Table 1). This tool was developed by analyzing 1.4 million genomic markers, including coding and non-coding RNA. The signature is based on 22 expressed RNA biomarkers involved in various biologic pathways (cell differentiation, proliferation, structure, adhesion and motility, immune modulation, cell-cycle progression, androgen signaling) [19]. The outcome of the test is represented by a derived continuous risk score—a genomic classifier (GC) ranging from 0 to 1. Each score has an associated probability of metastasis at 5 years postoperatively, with 1 being the highest risk. Specifically, Decipher™ has been studied and approved in the United States for PCa patients treated with RP and adverse pathology (pT3 and/or positive margins or BCR) to evaluate the risk of developing BCR or clinical progression (metastasis) during follow-up. However, although these recommendations have been reported in the National Comprehensive Cancer Network (NCCN) guidelines [20], clinical use and long-term data are needed to judge the real added value [16].

**Table 1:** Characteristics of studies evaluating Decipher™

First author and year	Number of cases	Study population	End point	Main results
Erho et al. [19] 2013	545 patients	Radical Prostatectomy	Metastases	Area Under Curve of 75 % in prediction of metastases
Karnes et al. [22] 2013	219 patients	Radical Prostatectomy	Metastases	Area Under Curve of 79 % in prediction of metastases
Den et al. [24] 2014	139 patients	Radical Prostatectomy and adjuvant radiation therapy	Biochemical recurrence and metastases	Area Under Curve of 78 % and 80 % in prediction of biochemical recurrence and metastases, respectively
Ross et al. [23] 2014	85 patients	Radical Prostatectomy	Metastases after biochemical recurrence	Area Under Curve of 82 % in prediction of metastases in patients that have developed Biochemical recurrence
Cooperberg et al. [28] 2015	185 patients	Radical Prostatectomy	Disease free survival	Predict high risk for PCa death
Den et al. [25] 2015	188 patients	Radical Prostatectomy and adjuvant radiotherapy in pT3 or positive margin patients	Metastases	Predict development of clinical metastases
Klein et al. [27] 2014	162 patients	Radical Prostatectomy for high risk prostate cancer in node positive patients	Metastases within 5 year after surgery	Predict development of metastases within 5 years after surgery
Alshalalfa et al. [21] 2015	463 patients	Radical Prostatectomy	Differences between patients that developed Biochemical recurrence or metastasis during follow up	Patients that develop metastases after BCR can be identified

##### Natural history after BCR in RP PCa patients

Alshalalfa et al. [21] analyzed the differences in patients that developed BCR, clinical metastases or were free from any adverse outcomes during follow-up, and found that Decipher™ can represent a useful tool to better prognosticate patients who develop metastasis after BCR. In this study, patients without evidence of disease and those with BCR-only were found with similar transcriptional profiles, in contrast to patients that developed metastases and showed a distinct transcriptional profile that can be detected in the primary tumor/RP specimen. These findings were validated in 219 high risk PCa patients treated at Mayo Clinic with RP. On multivariable analyses, higher GC scores were most prognostic for the development of metastases [22]. Similar results were observed by Ross et al. [23] with 85 high risk patients who developed BCR after RP. Moreover, with this natural history study, i.e. without secondary treatments potentially confounding the results, the GC score was able to differentiate between patients that eventually progress after BCR and those that do not, indicating perhaps the patients to earlier intervene on versus those that might continue to be followed.

##### Immediate versus delayed adjuvant radiotherapy after RP

Den et al. [24] studied patients treated by RP and adjuvant radiotherapy (aRT) for pT3 or positive surgical margin PCa, and found the area under the curve (AUC) predicted BCR and clinical metastases of 78 % and 80 %, respectively. Patients with a lower GC score on their RP specimen were those who may benefit from delayed RT at further BCR, in comparison to patients with higher GC who may benefit from aRT. In a follow-up study [25], 188 patients from both Thomas Jefferson University and Mayo Clinic treated with RP and adjuvant or salvage RT were evaluated with a median follow-up of 10 years. The GC score was able to best prognosticate the occurrence of metastases, and provided further support that patients with low GC scores can probably be treated with observation and salvage RT as needed, while patients with high GC scores could benefit the most from aRT. This is a step towards balancing the risks and benefits of delivering aRT, since all these male patients would have been advised to consider aRT based on their RP pathology, and evidence that the GC score is not only prognostic but potentially predictive in regards to the timing of postoperative RT. Furthermore, a study by Lobo et al. [26] showed a benefit in quality-adjusted life-years (QALYs) by using the Decipher™ test in the postoperative setting.

##### Combination of validated clinical and genomic risk stratification for prediction of survival after RP

An improvement in the prognostication of clinical outcomes after RP has been described by Klein [27] and Cooperberg [28] by including validated clinical parameters and Decipher™ scores in the same model. The AUC in prognostication of metastases was 77 %, 75 % and 72 % for Decipher™, and the commonly used clinical scores from the Stephenson model and Cancer of the Prostate Risk Assessment Score (CAPRA-S), respectively. However, when Decipher™ was integrated into the Stephenson nomogram, the AUC rose to 79 %. Similar results were presented for cancer-specific mortality (CSM) after RP; in fact, the AUCs were 75 % and 78 % for CAPRA-S and Decipher™, respectively. Patients with both high GC and CAPRA-S risk scores were at major risk for CSM. Moreover, Decipher™ was able to reclassify many patients stratified to high risk based on CAPRA-S to an actual lower risk based on a low GC, thus potentially sparing patients postoperative therapy, and at least providing less worry to the patients.

#### Oncotype DX®

Oncotype DX® is a test that has been developed by Genomic Health (Redwood City, CA, USA) for prostate cancer, and is well known for its prognostic and predictive capability in breast oncology. This tool is a quantitative RT-PCR assay performed on FFPE tissue from needle biopsies, incorporating 12 cancer-related genes representing four biological pathways (stromal response, androgen signaling, proliferation, cellular organization) and five reference genes, which are algorithmically combined to calculate the Genomic Prostate Score (GPS) [29]. The derived GPS ranges from 0 to 100, with the higher number correlating with a higher probability of harboring adverse pathology at RP in men diagnosed with low or low-intermediate risk prostate cancer at prostate biopsy (Table 2). Ostensibly, this test is utilized to counsel men regarding the best course of therapy (active surveillance vs. treatment) in newly diagnosed low or low-intermediate PCa. Specifically, NCCN guidelines recommend its utilization in post-biopsy decision-making for NCCN very low and low risk PCa at diagnosis with 10–20 years’ life expectancy [20].

**Table 2:** Characteristics of studies evaluating OncotypeDX®

First author and year	Number of cases	Study population	End point	Main results
Klein et al. [30] 2014	608 patients	Radical prostatectomy and Prostate biopsies	Metastases after radical prostatectomy and adverse pathology in radical prostatectomy specimen	Prediction of adverse pathology at radical prostatectomy using prostate biopsies tissue.
Cullen et al. [31] 2015	431 patients	Prostate biopsies in very low, low and intermediate Prostate cancer patients	Adverse radical prostatectomy pathology and biochemical recurrence	Increased BCR risk at univariable analyses and after adjusting for risk groups at multivariable using prostate biopsy tissue

##### Prediction of survival outcomes in low or intermediate risk PCa

Klein et al. [30] designed an initial study including 395 men who had low to intermediate risk prostate cancer who underwent RP. The biopsy-based 17-gene GPS improved on the prediction of the presence or absence of adverse pathology, defined as primary Gleason score pattern of 4 or any pattern of 5, or non-organ-confined disease. Cullen et al. [31] described 431 patients with NCCN very low, low and intermediate risk-stratified PCa at biopsy, and demonstrated the ability of Oncotype DX® on a biopsy specimen (largest tumor foci) to independently better predict adverse pathology at RP and consequently BCR than the risk group variables.

#### Prolaris®

Prolaris® is also a prognostic test developed by Myriad Genetics (Salt Lake City, UT, USA). This test is based on the expression of 31 cell-cycle progression (CCP) and 15 housekeeping genes (Table 3), and is an extension of their breast cancer test. The result is represented by a proliferative index, expressed as a CCP score [32]. The result has shown improved prognostic ability over clinicopathologic variables in various settings and tissues (biopsy and RP), and for various outcomes. This tool is used on biopsies from low and very low risk men to help better determine if immediate or deferred (conservatively managed) treatment is the better course. Again, like others, it is a prognostic assay and does not provide an answer to the treatment conundrums, but a probability of an event in the future, such as BCR and prostate cancer-specific mortality. In context, NCCN recommends its use in the post-biopsy period for NCCN very low and low risk PCa at diagnosis for patients with at least 10 years’ life expectancy [20].

**Table 3:** Characteristics of studies evaluating Prolaris®

First author and year	Number of cases	Study population	End point	Main results
Cuzick et al. [32] 2011	703 patients	Transurethral resection of prostate or radical prostatectomy	Biochemical recurrence and disease free survival	Predict biochemical recurrence and disease free survival
Cuzick et al. [33] 2012	349 patients	Prostate biopsies	Disease free survival	Predict disease free survival
Cooperberg et al. [36] 2013	413 patients	Radical prostatectomy	Biochemical recurrence	Predict biochemical recurrence
Freedland et al. [35] 2013	141 patients	Prostate biopsies in patients treated with EBRT	Biochemical recurrence and disease free survival	Predict disease free survival
Bishoff et al. [34] 2014	582 patients	Prostate biopsies	Biochemical recurrence and metastases	Predict biochemical recurrence and metastases

##### Prediction of survival outcomes in low or very low risk patients

Cuzick et al. [33] investigated 349 prostate biopsy samples evaluating the impact of CCP score—Prolaris®—on disease-free survival. At multivariable analysis, the CCP score showed a hazard ratio of 1.65, which was the strongest factor. Bishoff et al. [34] analyzed 582 patients from the Martini Clinic (*n* = 283), Durham Veterans Affairs Medical Center (*n* = 176) and Intermountain Healthcare (*n* = 123), and showed an association of the biopsy CCP score with adverse outcomes after surgery. In a larger study, Cuzick et al. [32] evaluated the CCP score and its ability of prognosticating BCR in a US cohort of RP patients and mortality in a UK cohort of patients treated with transurethral resection of the prostate (TURP). Additionally, the CCP was validated by Freedland et al. [35] in primary external radiation-treated patients. Cooperberg et al. [36] analyzed 413 RP patients with a CCP score in prognosticating BCR, and the best AUC came from a combined CCP score and CAPRA-S for the overall cohort and the low risk subset.

#### ProMark®

ProMark® is the newest commercially available biomarker panel and is a protein-based test developed by Metamark (Cambridge, MA, USA) as a quantitative multiplexed proteomics assay. Originally, there were 12 protein markers that demonstrated the best prognostication from a biopsy specimen [37]. Subsequently, the signature was refined and includes eight protein markers [38], with a score (0–1) reflecting the probability of adverse pathology at RP. To our knowledge, there is a lack of validation of this panel, most likely related to the more recent development.

##### Prediction of aggressiveness of prostate biopsy samples

Blume-Jensen et al. [38] found a favorable biomarker risk score (defined as ≤ 0.33) and a non-favorable risk score (defined as > 0.80) in 10 % and 5 % patients, respectively. The predictive value for non-favorable pathology was 76.9 % considering a biomarker risk score of > 0.80. At a biomarker risk score of ≤ 0.33 evaluating the prediction of favorable pathology disease, the predictive values were 95 %, 81.5 % and 87.2 % in NCCN very low and low risk, and low risk D’Amico groups, respectively [38].

#### ConfirmMDx

ConfirmMDx is a methylation marker genetic test developed by MDxHealth (Irvine, CA, USA). The test records a 90 % negative predictive value within 30 months of the initial biopsy, and has been reported as the most significant predictor of biopsy results [39]. The impact of this test on re-biopsy was recently assessed in 138 patients; among those with a negative ConfirmMDx assay, only 6 patients (4 %) underwent repeat biopsies [40]. ConfirmMDx may therefore be of value in the decision of repeat biopsies after a negative initial prostate biopsy.

### Other available tissue-based markers

#### Ki-67

Ki-67 is a nuclear protein associated with ribosomal RNA synthesis and has been typically measured by semi-quantitative immunohistochemistry (IHC) to assay cell proliferation in cancer. Although not tied to a commercial entity nor licensed, the IHC is typically performed using a MIB-1 antibody and reported as a percent of cells staining positively, which reflects their proliferation and correlates with various cancer outcomes, including PCa. Although several reports have assessed the impact of Ki-67 evaluation at the time of biopsy or after RP, at the present time no clinical use has been recommended by NCCN, which recently excluded the use of this test from the recommendation [20].

##### Prostate biopsies

The prognostic value of K1-67 in prostate needle biopsies are documented by several authors [41–43]. Tollefson et al. [44] most recently reported data of 451 consecutive patients with biopsy-proven PCa treated with RP at Mayo Clinic. The results confirmed that a combination of Gleason score, perineural invasion and Ki-67 expression is the most effective predictor of cancer outcome at the time of prostate needle biopsy. It is not known how this single marker of cell proliferation compares to the CCP. Ki-67 staining index on biopsies has been tested on 573 patients treated with PCa radiotherapy with a median follow-up of 96 months [45]. The Ki67 staining index was the most significant determinant of distant metastases and cancer-specific mortality during follow-up.

##### Outcomes after radical prostatectomy for localized PCa

Ki-67 staining index has also been reported to be a powerful predictor of survival after RP [42, 46, 47]. Furthermore, Mathieu et al. [48] analyzed data of 3,123 patients who underwent RP and confirmed that Ki-67 is a strong prognostic variable for BCR, and might be informative in clinical decision-making regarding adjuvant therapy and optimizing follow-up schedules.

#### PTEN

Considering mutations that have a well-established role in PCa, dysregulation of PTEN seems to be involved more frequently than others in advanced, localized or metastatic PCa, and has shown a prognostic impact [49–52]. PTEN is located on chromosome 10 and its mutation is considered a part of the PI3K/AKT signaling pathway and functions as a tumor suppressor gene. PTEN appears to be the most frequently mutated tumor suppressor gene and deletion of PTEN has been recorded in 10–70 % of RP specimens [53–57], with the variability likely related to the risk group, with the higher the risk the higher likelihood of a PTEN deletion. A recent publication by Murphy et al. [58] demonstrated that PTEN loss is infrequent in clinically insignificant PCa (Gleason score 6 and low volume) and is associated with higher-grade tumors. This finding is also supported by others whether on biopsy [59, 60] or RP specimens [49, 61] and in castration-resistant tumors [62]. Again, it is unknown how a single gene may perform in relation to an aforementioned panel and what the prognostic differences would be. The costs of a single gene assay should be cheaper than a panel, but given the heterogeneity of PCa in terms of foci and different biologic pathways there is an obvious potential to not completely capture what is needed with a single marker. As per Ki-67 marker, its use has been excluded from the clinical recommendation by NCCN [20].

## Conclusions

Available genomic assays have improved the prognostic ability over clinicopathologic parameters of localized PCa. Ideally, these assays should be prospectively applied, or even retrospectively applied to prospective studies, to further validate their clinical utility in prognostication and even prediction in terms of what treatment should be applied either at a new diagnosis or post-RP. In addition to their clinical value, more work is needed in regards to their financial impact on the cost of localized PCa care.
